# Toxicity Profiles of Systemic Therapies for Advanced Hepatocellular Carcinoma: A Systematic Review and Meta-analysis

**DOI:** 10.1001/jamanetworkopen.2022.22721

**Published:** 2022-07-18

**Authors:** Christopher D. Griffiths, Betty Zhang, Kasia Tywonek, Brandon M. Meyers, Pablo E. Serrano

**Affiliations:** 1 Department of Surgery, McMaster University, Hamilton, Ontario, Canada; 2 Department of Anesthesia, University of Ottawa, Ottawa, Ontario, Canada; 3 Department of Oncology, McMaster University, Hamilton, Ontario, Canada; 4 Department of Health Research Methods, Evidence, and Impact, McMaster University, Hamilton, Ontario, Canada; 5 Division of General Surgery, Juravinski Hospital, Hamilton, Ontario, Canada

## Abstract

**Importance:**

The recent development of targeted therapy and immunotherapy has made neoadjuvant therapy an attractive option for patients with hepatocellular carcinoma (HCC). However, surgeons are concerned that adverse effects of neoadjuvant therapy with these agents could lead to delayed or even cancelled surgeries.

**Objective:**

To summarize the current evidence regarding toxicity profiles for tyrosine kinase inhibitors (TKIs) and immune checkpoint inhibitors (ICIs) among patients with HCC.

**Data Sources:**

Medline, Embase, and Cochrane Central Register of Controlled Trials (CENTRAL) were searched from January 1990 and December 2021.

**Study Selection:**

Single-group, placebo-controlled, and dual-agent clinical trials comparing TKIs and ICIs in patients with HCC were eligible for inclusion.

**Data Extraction and Synthesis:**

Following the Preferred Reporting Items in Systematic Reviews and Meta-analysis guideline, 2 reviewers independently extracted data. A random-effects model was used.

**Main Outcomes and Measures:**

The primary outcome was the proportion of patients with clinically significant liver-related adverse events. Secondary outcomes included the proportion of patients who experienced clinically relevant (grade 3 or higher) adverse events and significant adverse events (ie, those that were life threatening, required hospitalization, or prolonged disability) as well as the risk ratio (RR) of these complications.

**Results:**

Overall, 30 studies with 12 921 patients were included. Patients had a mean (range) age of 62 (18-89) years; a mean (SD) 84% (3) were male; a mean (SD) 82% (16) had Barcelona Clinic Liver Cancer stage C HCC; and a mean (SD) 97% (6) had Childs A cirrhosis. Overall, 21% (95% CI, 16%-26%) of patients receiving TKIs had liver toxic effects compared with 28% (95% CI, 21%-35%) of patients receiving ICIs. Severe adverse events occurred in 46% (95% CI, 40%-51%) of patients receiving TKIs compared with 24% (95% CI, 13%-35%) of patients receiving ICIs. Compared with patients receiving sorafenib, other TKIs were associated with similar rates of liver toxic effects (RR, 1.06; 95% CI, 0.92-1.24) but higher rates of severe adverse events (RR, 1.24; 95% CI, 1.07-1.44). Comparing ICIs with sorafenib, there were similar rates of liver toxic effects (RR, 1.10; 95% CI, 0.86-1.40) and severe adverse events (RR, 1.19; 95% CI, 0.95-1.50).

**Conclusions and Relevance:**

In this systematic review and meta-analysis, serious adverse events were lower with ICIs than with TKIs, while liver toxic effects were similar. Combination therapy with novel ICIs is an appealing option in trials of neoadjuvant therapy for patients with HCC, requiring evaluation in preoperative trials.

## Key Points

**Question:**

What are the adverse effect profiles of immune checkpoint inhibitors (ICIs) and tyrosine kinase inhibitors (TKIs) in the treatment of advanced hepatocellular carcinoma (HCC)?

**Findings:**

In this systematic review and meta-analysis of 30 prospective clinical trials including 12 921 patients, ICIs were associated with similar rates of liver toxic effects but improved rates of severe adverse events compared with TKIs.

**Meaning:**

In this study, ICIs had a more favorable toxicity profile than TKIs among patients with advanced HCC and therefore may be the more appropriate agent in the neoadjuvant setting.

## Teaser

This systematic review and meta-analysis summarizes the current evidence regarding toxicity profiles for tyrosine kinase inhibitors (TKIs) and immune checkpoint inhibitors (ICIs) among patients with hepatocellular carcinoma (HCC).

## Introduction

Hepatocellular carcinoma (HCC) is the second most common cause of cancer-related deaths worldwide.^1^ The current global estimate of more than 900 000 new cases being diagnosed every year is expected to increase, specifically in Western countries owing to increase in nonalcoholic steatohepatitis.^1,2^ Unfortunately, most cases of HCC are unresectable at diagnosis and therefore treated with palliative, rather than curative, intent.^3^ For patients diagnosed with advanced disease, including extrahepatic spread, vascular invasion, and tumor-related symptoms, systemic therapy is recommended.^4,5^ Over the last 10 years, new options for systemic therapy—and the survival benefits offered by these therapies—have increased significantly. The success of these treatments is largely because of their ability to modulate the patient’s immune system, allowing for endogenous tumor recognition to combat further tumor growth. For HCC, these therapies include both small-molecule tyrosine kinase inhibitors (TKIs) and immune checkpoint inhibitors (ICIs).^3,5^

In HCC, therapies typically target multiple growth factors such as the RAF/MEK/ERK pathway or more specific vascular endothelial growth factor receptor, platelet-derived growth factor receptor, and KIT.^6^ Approved TKIs include sorafenib, lenvatinib, regorafenib, and cabozantinib.^7,8,9,10,11^ ICIs typically target proteins such as the programmed cell death receptor 1 (PD-1), programmed cell death receptor ligand 1 (PD-L1), and cytotoxic T lymphocyte–associated antigen 4 (CTLA-4). Tumor cells manage to evade the body’s immune system by upregulating so-called immune checkpoints and have therefore become a common target of anticancer therapy by reversing this T-cell inhibition.^6^ In HCC, these therapies include but are not limited to nivolumab, pembrolizumab, ipilimumab, cemiplimab, and atezolizumab.^12,13,14^ However, ICIs are associated with immune-related adverse events that include toxic effects of the skin, bowel, and liver.^15,16^

As HCC therapy evolves to combine medical therapy with ICIs and TKIs and targeted surgical and locoregional therapies, such as ablation and embolization, clinicians require a more comprehensive understanding of how treatment toxic effects affect subsequent therapies. This study sought to compile the current evidence of toxic effects, especially hepatotoxic effects related to systemic therapy for HCC, with an eye toward designing clinical trials for neoadjuvant treatment of HCC.

## Methods

### Literature Search

This study followed the methods found in the Cochrane Handbook for Systematic Reviews of Interventions and was reported according to Preferred Reporting Items for Systematic Reviews and Meta-analyses (PRISMA) guidelines.^17,18^ Databases Medline, Embase, and Cochrane Central Registers of Control Trials (CENTRAL) were queried for studies published between January 1990 and December 2021. The search strategy included controlled vocabulary appropriate for each database (medical subject headings and Embtree) with additional keywords added to increase sensitivity. The Cochrane Review “Highly Sensitive Search Strategy for Identifying Randomized Trials” was used to search both MEDLINE and Embase. Manual searches of gray literature were conducted by going through relevant conference proceedings and reference lists of key articles. There were no language restrictions imposed, and articles were translated to English when necessary. To ensure a comprehensive search, experts were consulted to identify any relevant articles that were missing.

### Study Selection

Single-group, placebo-controlled, and dual-agent clinical trials in phase 2 and 3 for HCC were included. Included therapies were TKI, PD-1/PD-L1, and CTLA-4 inhibitors as well as any combination of these therapies. When only an abstract could be found, authors were contacted to provide additional relevant information. The primary outcome was the proportion of patients who had clinically significant liver-related adverse events, and secondary outcomes included the proportion of patients who experienced clinically relevant (grade 3 or higher) adverse events and significant adverse events (ie, those that were life threatening, required hospitalization, or prolonged disability) as well as the risk ratio of these complications.^19^

Two independent reviewers were involved in the selection and reading of abstracts and full-length papers to independently establish whether the eligibility criteria for inclusion were met. Prior to screening, detailed eligibility criteria were established and used by both reviewers. The data collected from Medline, Embase, and CENTRAL were exported to EndNote version X9 software (Clarivate) to detect any title and abstract duplicates. Reviewers each received a copy of the final list generated and distributed via Distiller SR web-based software to facilitate the inclusion and exclusion process for each study. A PRISMA diagram was generated to organize all relevant article information from the screening process. Once abstract screening was complete, the full text of all pertinent references was obtained. For any articles for which there was discrepancy between reviewers, in-depth analysis was conducted to determine the reason behind each reviewer’s choice. If consensus could not be reached, a third-party reviewer was consulted to adjudicate a final decision. Cohen κ interrater reliability coefficient was used to assess the agreement level between reviewers on the included articles.

### Data Extraction, Risk of Bias, and Quality of Evidence

Data extraction from included studies was performed independently by each reviewer using a standardized form. Data collected included study design, patient characteristics, treatment category, incidence of planned surgery, and incidence of clinically relevant adverse events. Incidence was collected as proportions. Risk of bias was assessed independently by the 2 reviewers using the Cochrane Risk of Bias Tools for randomized trials and the Methodological Index for Nonrandomized Studies (MINORS) tool for single-group clinical trials.^20,21^ After independent assessment, any disagreements were resolved by consensus. The Grading of Recommendations, Assessment, Development and Evaluation (GRADE) approach recommended by Cochrane Collaboration was used by the reviewers to assess the confidence in the estimates of effect.^22^

### Statistical Analysis

The proportion of patients who experienced grade 3 or 4 adverse events, specifically gastrointestinal, lung, and liver events, after either immunotherapy or TKI monotherapy or any combination therapy was collected as well as the associated standard error. Results were compiled and forest plots generated using Review Manager version 5.0 to calculate the pooled proportion of patients who experienced each subset of adverse events. Subgroup analyses were used to compare the proportion of patients who reported adverse events among the different treatment types (CTLA-4, PD-1/PD-L1, TKI, or combination). Pooled estimates were reported with a corresponding 95% CI and *P* value when applicable. The risk ratio (RR) of adverse events was also calculated and displayed on forest plots for all trials that had a comparator arm, including active agent treatment of placebo. A 2-sided *P* < .05 was considered statistically significant.

## Results

### Study Characteristics

**Figure 1.**
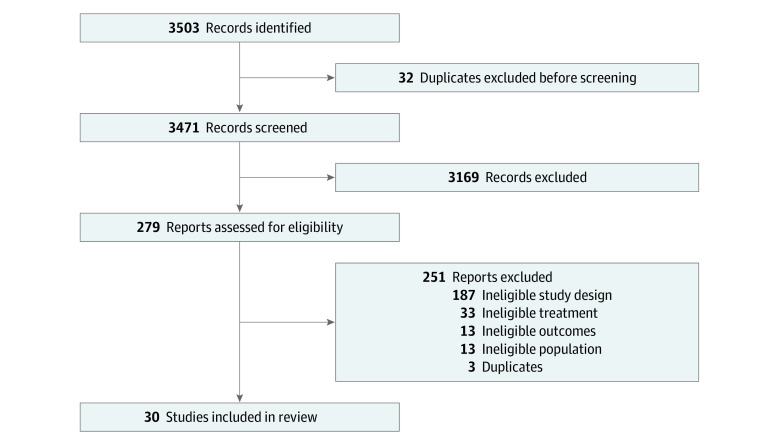
Study Flow Diagram

A total of 3503 relevant citations were identified, 30 of which met inclusion criteria (Table).^7,8,9,10,11,12,13,14,23,24,25,26,27,28,29,30,31,32,33,34,35,36,37,38,39,40,41,42,43,44^ Study selection revealed Cohen κ interrater reliability of 0.96. A study flow diagram of study selection is illustrated in Figure 1. Studies included 18 phase 3 randomized trials,^7,8,9,10,11,12,13,14,23,24,29,34,36,39,41,42,43,44^ 10 phase 2 randomized trials,^25,26,27,30,31,32,33,35,38,40^ and 2 single-group phase 2 trials,^28,37^ conducted from 2008 to 2022. Three studies were abstract only.^25,31,35^ There were 25 studies^7,8,9,10,11,12,13,23,24,25,26,27,29,30,33,34,35,36,38,39,40,41,42,43,44^ that included a TKI in the treatment group, whereas 9 studies^12,13,14,26,28,31,32,35,37^ included an ICI. In total, these studies included 12 921 patients with advanced HCC: 9142 receiving a TKI, 1290 receiving ICI, 604 receiving both TKI and ICI, and 2084 receiving placebos.

**Table.** Study Characteristics

Source	Study type	Group 1		Group 2		Age, median (range), y	Patients, %			
		Agent	No.	Agent	No.		Male	Extrahepatic	BCLC C	Childs A
Abou-Alfa et al,^11^ 2018	Phase 3 RCT	Carbozantinib	470	Placebo	237	64 (22-86)	82	78	NA	100
Bruix et al,^10^ 2017	Phase 3 RCT	Regorafinib	374	Placebo	193	63 (IQR, 54-71)	88	72	87	98
Cainap et al,^23^ 2015	Phase 3 RCT	Linifanib	514	Sorafenib	521	59 (21-84)	86	58	82	93
Cheng et al,^7^ 2009	Phase 3 RCT	Sorafenib	150	Placebo	76	51 (23-86)	85	68	95	97
Cheng et al,^24^ 2013	Phase 3 RCT	Sunitinib	530	Sorafenib	544	59 (18-85)	83	77	85	99
Cheng et al,^25^ 2015	Phase 3 RCT; abstract	Nintedanib	63	Sorafenib	32	NA	NA	NA	NA	NA
Cheng et al,^26^ 2015	Phase 2 RCT	Tigatuzamab with sorafenib	108	Sorafenib	55	63 (27-84)	83	NA	98	100
Cheng et al,^27^ 2016	Phase 2 RCT	Dovitinib	82	Sorafenib	83	56 (27-83)	85	NA	98	100
Finn et al,^14^ 2020	Phase 3 RCT	Pembrolizumab	278	Placebo	135	66 (18-91)	82	70	79	99
Finn et al,^13^ 2020	Phase 3 RCT	Atezolizumab with bevacizumab	336	Sorafenib	165	65 (IQR, 56-71)	82	60	82	100
Finn et al,^28^ 2020	Phase 1b RCT	Lenvatinib with pembrolizumab	104	NA	NA	66.5 (47-86)	81	52	62	71
Johnson et al,^29^ 2013	Phase 3 RCT	Brivanib	577	Sorafenib	578	60 (25-89)	84	50	78	92
Kang et al,^30^ 2015	Phase 2 RCT	Axitinib	134	Placebo	68	62 (25-84)	82	70	80	100
Kaseb et al,^31^ 2020	Phase 2 RCT; abstract	Nivolumab	13	Nivolumab with ipulimamab	12	NR (32-83)	75	0	NA	100
Kelley et al,^32^ 2021	Phase 1/2 RCT	Durvulamab with tremulimumab	159	Durvulamab or tremulimumab	173	63.5 (26-89)	86	59	71	98
Koeberle et al,^33^ 2016	Phase 2 RCT	Everolimus with sorafenib	60	Sorafenib	46	65 (32-83)	83	55	73	84
Kudo et al,^34^ 2011	Phase 3 RCT	Sorafenib	229	Placebo	329	69 (29-86)	75	0	NA	NA
Kudo et al,^9^ 2018	Phase 3 RCT	Lenvatinib	478	Sorafenib	476	62 (20-88)	84	61	79	99
Lee et al,^35^ 2019	Phase 2 RCT; abstract	Atezolizumab with bevacizumab	60	Atezolizumab	59	NA	NA	NA	NA	NA
Llovet et al,^8^ 2008	Phase 3 RCT	Sorafenib	299	Placebo	303	Mean (SD), 62 (11.2)	87	52	83	98
Llovet et al,^36^ 2013	Phase 3 RCT	Brivanib	263	Placebo	132	63 (19-89)	84	65	86	92
Marron et al,^37^ 2022	Phase 2 RCT	Cemiplimab	21	NA	NA	68 (45-82)	86	0	14	100
Palmer et al,^38^ 2018	Phase 1/2 RCT	Nintedanib	62	Sorafenib	31	66 (28-86)	80	66	73	99
Rimassa et al,^39^ 2018	Phase 3 RCT	Tivantinib	226	Placebo	114	66 (19-87)	90	58	80	95
Santoro et al,^40^ 2013	Phase 2 RCT	Tivantinib	71	Placebo	36	69 (27-85)	81	66	NA	97
Yau et al,^12^ 2020	Phase 3 RCT	Nivolumab	371	Sorafenib	372	NA	NA	NA	NA	NA
Zhu et al,^41^ 2014	Phase 3 RCT	Everolimus	362	Placebo	184	66 (21-87)	85	74	86	98
Zhu et al,^42^ 2015	Phase 3 RCT	Ramucirumab	283	Placebo	282	63 (25-87)	84	72	88	98
Zhu et al,^43^ 2015	Phase 3 RCT	Erlotinib with sorafenib	362	Sorafenib with placebo	358	60 (NR)	80	59	85	100
Zhu et al,^44^ 2019	Phase 3 RCT	Ramucirumab	197	Placebo	95	64 (IQR, 56-73)	81	73	81	100

Abbreviations: BCLC, Barcelona Clinic Liver Cancer; NA, not applicable; NR, not reported; RCT, randomized clinical trial.

### Demographic Characteristics

The median age was 62 years (range, 18-89 years; IQR unavailable), with a mean (SD) of 84% (3) male participants, 61% (16) patients with extrahepatic disease, and 28% (16) patients with macrovascular invasion. Following Barcelona Clinic Liver Cancer staging, a mean (SD) of 82% (16) of included patients had stage C disease. Regarding liver disease, a mean (SD) of 97% (6) had Childs A cirrhosis. Two studies included patients with resectable HCC,^31,37^ while the remainder were considered advanced HCC (ie, not eligible for surgery).

### Liver-Related Adverse Events

**Figure 2.**
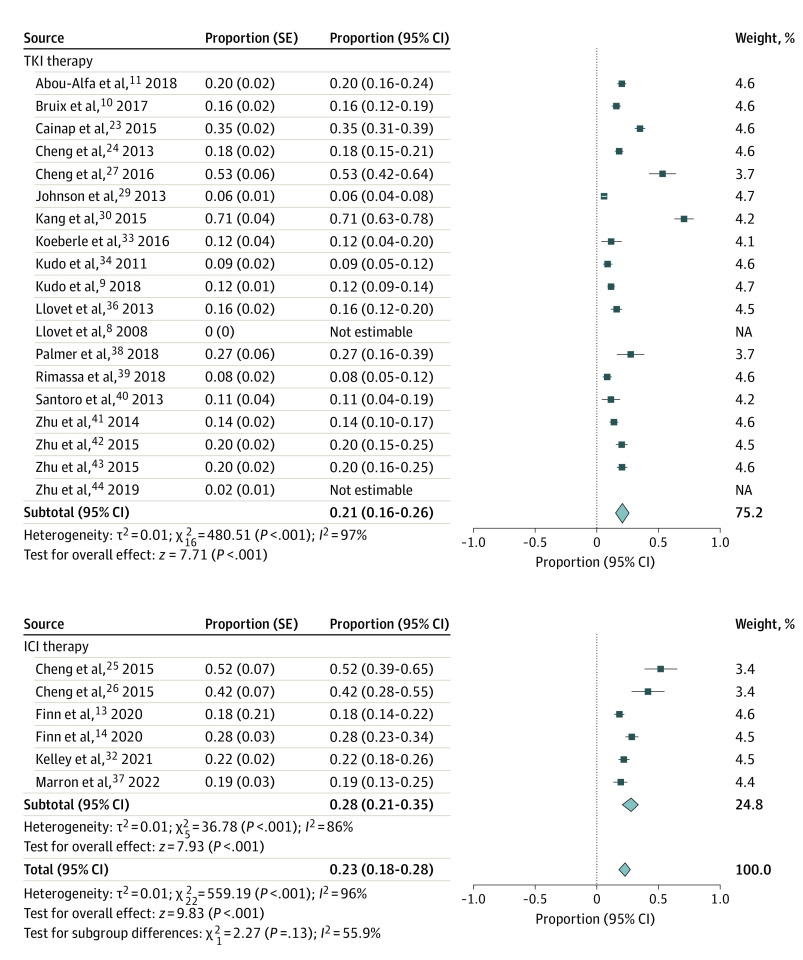
Proportion of Patients With Liver-Related Toxic Effects ICI indicates immune checkpoint inhibitors; and TKI, tyrosine kinase inhibitors.

Nineteen studies^8,9,10,11,23,24,27,29,30,33,34,36,38,39,40,41,42,43,44^ reported liver-related toxic effects for patients receiving a TKI, revealing a proportion of 21% (95% CI, 16%-26%) (Figure 2). In the 6 studies^13,14,25,26,32,37^ evaluating liver-related toxic effects for patients receiving an ICI, the proportion of liver toxic effects was 28% (95% CI, 21%-35%).

Eleven studies^8,10,11,30,34,36,39,40,41,42,44^ examined liver-related toxic effects, comparing TKI therapy with placebo (eFigure 1A in the Supplement). These studies found an RR of 1.38 (95% CI, 1.06-1.79) comparing 2897 patients receiving TKI with 1865 receiving placebo. In 1 study^14^ comparing ICI (n = 278) with placebo (n = 135), the KEYNOTE240 trial, there was an RR of 1.83 (95% CI, 1.18-2.82) for liver-related toxic effects.

Twelve studies^9,13,23,24,25,26,27,29,33,35,38,39^ compared liver-related toxic effects between patients receiving different agents (eFigure 1B in the Supplement). Compared with patients receiving sorafenib (n = 2634), patients receiving other TKIs (n = 2655) had an RR of 1.06 (95% CI, 0.92-1.24) for liver-related toxic effects. One smaller study^35^ compared patients receiving ICI with TKI (n = 60) vs ICI alone (n = 58) (atezolizumab with or without bevacizumab) and found no significant difference in liver-related toxic effects (RR, 1.29; 95% CI, 0.30-5.51). Similarly, comparing ICIs (n = 443) with sorafenib (n = 275), there were no significant differences (RR, 1.10; 95% CI, 0.86-1.40).^13,25,26^

### Severe Toxic Effects

**Figure 3.**
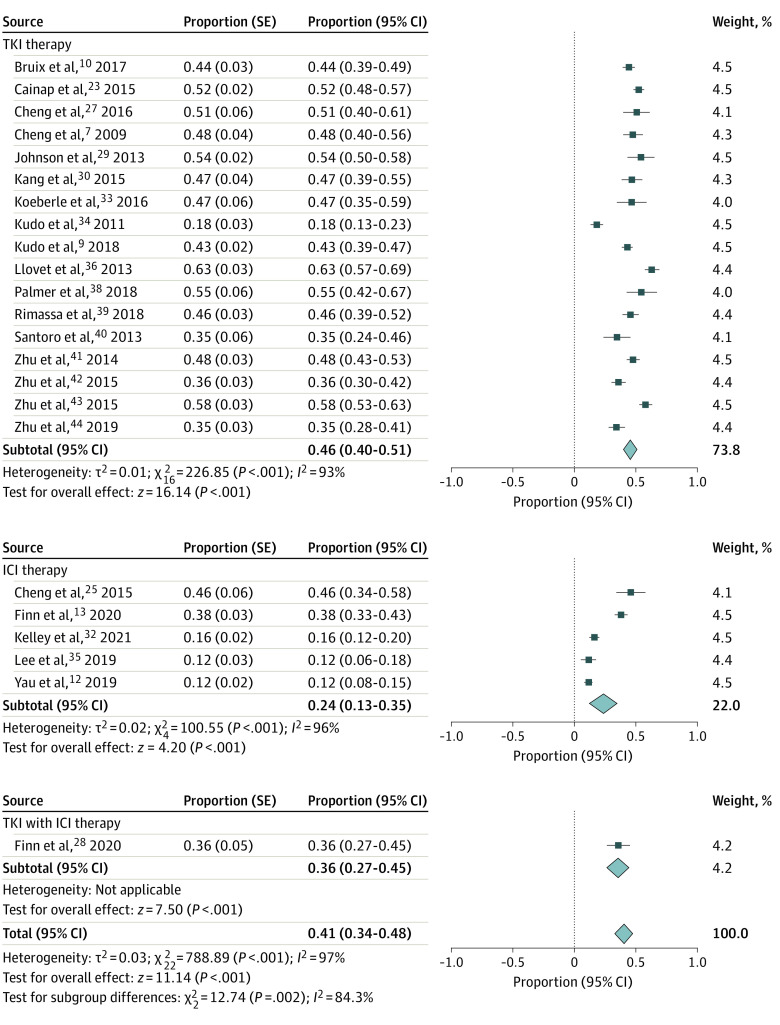
Proportion of Patients With Serious Adverse Events ICI indicates immune checkpoint inhibitors; and TKI, tyrosine kinase inhibitors.

The pooled proportion of patients undergoing therapy with severe adverse events was 41% (95% CI, 34%-48%). Overall, 46% (95% CI, 40%-51%) of patients receiving a TKI had a serious adverse event compared with 24% (95% CI, 13%-35%) of patients receiving an ICI and 36% (95% CI, 27%-45%) of patients receiving both a TKI and an ICI (Figure 3).

Severe toxic effects were evaluated in 11 studies^7,10,30,34,36,39,40,41,42,43,44^ comparing 2639 patients receiving TKI therapy with 1937 patients receiving placebo (eFigure 2A in the Supplement). These studies found an RR of 1.24 (95% CI, 1.07-1.44) for severe adverse events. There were no placebo-controlled trials with ICIs examining severe adverse events.

Six studies^9,23,24,25,27,29^ compared 1695 patients receiving other TKIs with 1684 patients receiving sorafenib (eFigure 2B in the Supplement). Rates of severe adverse events were higher in patients receiving other TKIs (RR, 1.24; 95% CI, 1.07-1.44). In 2 trials^12,13^ comparing 700 patients receiving ICIs with 528 patients receiving sorafenib, rates of severe adverse events were similar (RR, 1.19; 95% CI, 0.95-1.50). Two studies^33,43^ compared 238 patients receiving a second TKI and sorafenib with 209 receiving sorafenib alone, and severe adverse events were similar between groups (RR, 1.17; 95% CI, 0.85-1.61). Pooling all studies, active comparator agents had a higher rate of severe adverse events than sorafenib alone (RR, 1.21; 95% CI, 1.09-1.34).

### Grade 3 or Greater Adverse Events

**Figure 4.**
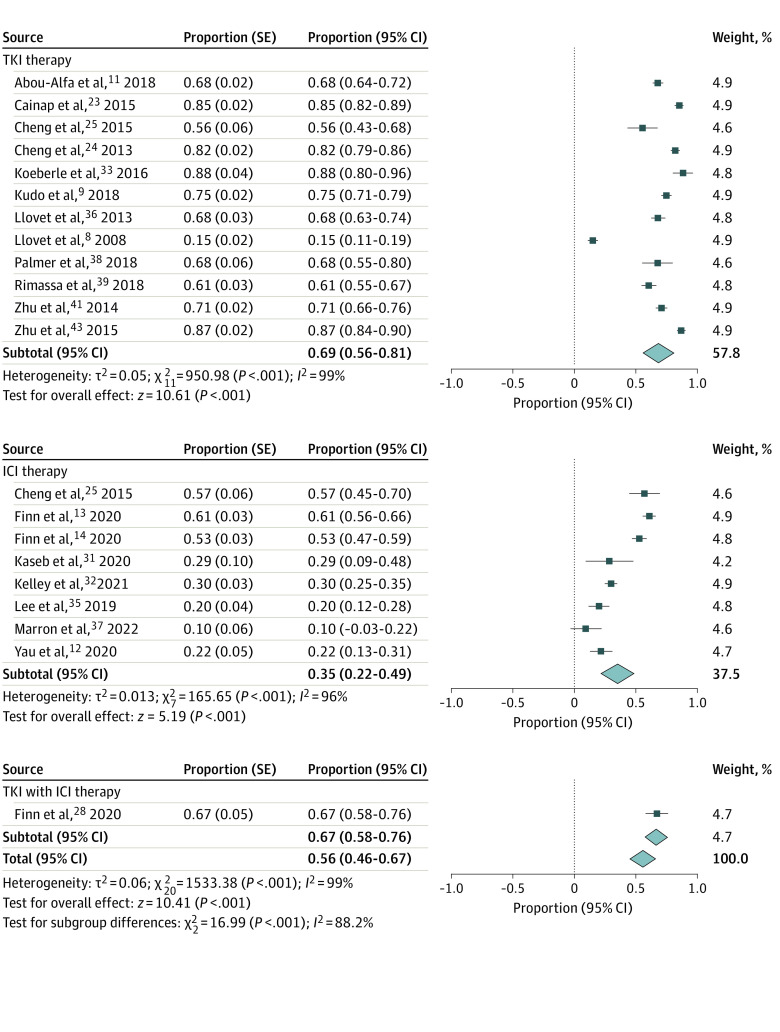
Proportion of Patients With Grade 3 or Higher Adverse Events ICI indicates immune checkpoint inhibitors; and TKI, tyrosine kinase inhibitors.

The pooled proportion of patients undergoing therapy with grade 3 or greater adverse events was 56% (95% CI, 46%, 67%). Overall, 69% (95% CI, 56%-81%) of patients receiving a TKI had a serious adverse event compared with 35% (95% CI, 22%-49%) of patients receiving an ICI and 67% (95% CI, 58%-76%) of patients receiving both a TKI and an ICI (Figure 4).

Some studies did not report severe toxic effects, but rather grade 3 or higher adverse events. In 5 placebo-controlled trials,^8,11,36,39,41^ 1615 patients receiving TKIs were compared with 968 patients receiving placebo, with an RR of 1.65 (95% CI, 1.27-2.14) for adverse events grade 3 or higher (eFigure 3A in the Supplement). In 1 placebo-controlled trial,^14^ pembrolizumab was given to 278 patients compared with 135 who received placebo, with an RR for adverse events of grade 3 or higher of 1.15 (95% CI, 0.93-1.43).

Six studies^23,24,25,34,38,43^ with an active comparator evaluated grade 3 or higher adverse events in 1999 patients receiving a TKI compared with 1949 receiving sorafenib (eFigure 3B in the Supplement). Grade 3 or higher adverse events were similar with an RR of 1.07 (95% CI, 0.92-1.24). The IMbrave150 trial^13^ compared 329 patients receiving atezolizumab and bevacizumab with 156 patients receiving sorafenib. Grade 3 or higher adverse events were similar between groups with an RR of 1.00 (95% CI, 0.86-1.17). One study compared 60 patients receiving everolimus plus sorafenib with 46 patients receiving sorafenib alone, with an RR of 1.23 (95% CI, 1.00-1.51) for grade 3 or higher adverse events.^33^

### Risk of Bias and Quality of Evidence

A total of 28 included studies were phase 2 and phase 3 randomized clinical trials, and 2 were single-group prospective phase 2 trials. While 16 trials^7,8,10,11,13,14,29,30,34,36,39,40,41,42,43,44^ were double-blind, 12 trials^9,12,23,24,25,26,27,31,32,35,38^ were open label, introducing a possibility for reporting bias (eFigure 4 in the Supplement). The 2 single-group studies^28,37^ scored 14 of 16 and 16 of 16 on MINORS (eTable in the Supplement). The direction of bias was unclear. Regarding quality of evidence, GRADE determined a moderate level of evidence for these prospective and randomized clinical trials (eFigure 5 in the Supplement). Adverse events were coded and detected according to the same criteria in each study. The major difference in population across trials was the degree of pretrial treatment, with inconsistencies in those who had already received locoregional therapies or first-line chemotherapy, such as sorafenib, and the directness of evaluation.

## Discussion

For patients with advanced HCC, TKIs and ICIs have improved survival in the absence of surgical candidacy; their role, however, in the neoadjuvant setting has not yet been well tested. This systematic review examined the relative safety profile of these classes of therapy in terms of toxic effects. Examining 30 studies and 12 921 patients, we found that a greater proportion of patients treated with TKIs had serious adverse events than those treated with ICIs, although liver-related toxic effects were similar between therapies.

Neoadjuvant therapy offers the opportunity to administer potentially important therapy prior to surgery, avoiding delays related to postoperative complications; it can also be used to downstage advanced disease to resectable disease and ensure appropriate patient selection, both in terms of tumor biology and patient factors.^45,46^ In HCC, this is partially complicated because most patients have cirrhosis and need an adequate functional liver remnant. A few studies have explored the use of neoadjuvant locoregional treatments in HCC. A systematic review by Qi et al^47^ examined 55 studies with preoperative and postoperative transarterial chemoembolization (TACE) for resectable HCC, finding no significant difference in disease-free or overall survival.^47^ These studies had significant heterogeneity in the chemotherapy agent used and did not evaluate TACE as a downstaging mechanism. Similarly, for HCC patients on the waiting list for liver transplantation, weak evidence suggests locoregional therapies may be used for downstaging or to maintain candidacy beyond 6 months.^48,49,50^ The ability of embolization to expand the functional liver remnant is appealing but perhaps counteracted by the portal venous spread of HCC and its propensity for multifocal recurrence.^51,52,53^ ICIs have recently been proposed as pretransplantation therapy, either as a bridge or downstaging; however, an early case report raised concerns for rejection following pretransplantation nivolumab administration.^54^ More recently, a case series of 9 patients with HCC treated with nivolumab before transplantation had no severe adverse outcomes following the procedure, promoting optimism for ICIs in the pretransplantation setting.^55^ The natural biology of HCC therefore suggests that if systemic therapy can treat resectable tumors or downsize existing tumors to resectable disease without significant toxic effects, while simultaneously preventing hepatic spread, it could serve as neoadjuvant therapy.^45^

The only evidence for preoperative sorafenib has been in the neoadjuvant setting for liver transplantation.^46^ In 1 study^56^ of 33 listed patients awaiting transplant for HCC, 10 patients received sorafenib and had higher rates of biliary complications compared with the control group (67% vs 17%; *P* = .01). Furthermore, there was a nonstatistically significant increase in postoperative death following exposure to sorafenib (20% vs 9%; *P* = .56).^56^ Another randomized clinical trial^57^ examined TACE with sorafenib vs TACE alone in 50 patients awaiting transplantation. In this study,^57^ sorafenib was associated with increased rates of severe adverse events (50% vs 16%). The ICI nivolumab was used in a 21-patient trial^31^ as neoadjuvant therapy to surgical resection, with 21 of 27 patients reaching surgery following nivolumab with or without ipilimumab therapy.^31^ Another study included in our meta-analysis,^37^ a single-arm phase 2 trial examining the ICI cemiplimab in the neoadjuvant setting for resectable HCC, found that 20 of 21 patients went on to undergo surgical resection. Furthermore, 35% of patients in this trial^37^ and 40% in the nivolumab trial^31^ had at least partial pathologic response, suggesting downstaging could be possible. Considering the results of our meta-analysis, which suggest that ICIs have similar toxic effects as sorafenib but lower toxic effects compared with other TKIs, the use of novel immunotherapies may prove to be safe in the neoadjuvant setting, as evidenced by recently started neoadjuvant trials in HCC.^58,59,60^

Neoadjuvant therapy may help to determine which patients are the best candidates for surgical resection in terms of selecting those patients with good objective response to therapy. Evaluating existing studies, current American Society of Clinical Oncology guidelines consider atezolizumab and bevacizumab to be first-line therapy for advanced HCC and lenvatinib to be equivalent to sorafenib.^5^ In the IMbrave150 trial^13^ comparing atezolizumab and bevacizumab with sorafenib in advanced HCC, combination therapy with atezolizumab and bevacizumab led to a 27% objective response rate according to mRECIST, compared with 12% with sorafenib (*P* < .001). Furthermore, with combination therapy in the IMbrave150 trial^13^ there was no significant difference in severe adverse events. Lenvatinib, in a similar 2018 trial by Kudo et al,^9^ led to improved objective response rate according to mRECIST, compared with sorafenib (24% vs 9%; *P* < .001). Additionally, adjusting for treatment duration, severe adverse events per patient-year were similar between groups (3.3 vs 3.2 episodes per patient-year).^9^ Focusing on these 2 therapies (atezolizumab and bevacizumab or lenvatinib alone) would be reasonable neoadjuvant therapy agents in future trials based on improved response rates and similar toxic effects when compared with sorafenib in the limited available head-to-head data.

### Limitations

This study has limitations. This was a large systematic review and meta-analysis of level-1 evidence featuring more than 12 000 patients, the vast majority with Barcelona Clinic Liver Cancer stage C advanced HCC and Childs A cirrhosis. While these trials are well-designed with consistent dosing, the reporting of adverse events varied between studies, with not all studies reporting hepatotoxic effects and some studies reporting severe adverse events, while others reported grade 3 or greater. Furthermore, particularly in the placebo-controlled studies not including sorafenib, inclusion criteria varied, with some studies including only patients who had progression on sorafenib, which may contribute to seemingly worse toxic effects; however, this was less of a factor for trials that included sorafenib as a treatment, which provided the bulk of our analysis. Overall, the heterogeneity in pretrial treatment is the most significant confounding factor not well accounted for in this study.

## Conclusions

For patients with advanced HCC, systemic therapy has evolved over the past ten years to offer a survival benefit with both TKIs and ICIs. Despite this, converting unresectable disease to disease amenable to surgical resection has not yet been achieved. We found that ICIs were associated with similar toxic effects as sorafenib but improved compared with other TKIs. When considering objective response rates, combination therapy with atezolizumab and bevacizumab or lenvatinib alone likely offer the most promise in the neoadjuvant setting in terms of objective response and toxic effects without preventing patients from reaching surgery.

## Notes

Supplement. **eFigure 1. **Liver-Related Toxic Effects in Placebo-Controlled Trials and Trials With Active Comparator **eFigure 2. **Serious Adverse Events in Placebo-Controlled Trials and in Trials With Active Comparator **eFigure 3. **Grade 3 or Higher Adverse Events in Placebo-Controlled Trials and Trials With Active Comparator **eFigure 4. **Risk of Bias 2 Assessment **eTable.** MINORS Assessment **eFigure 5. **GRADE of Evidence Assessment
