# Supplementary material for: Toxicity Profiles of Systemic Therapies for Advanced Hepatocellular Carcinoma: A Systematic Review and Meta-analysis
Source: JAMA Netw Open. 2022 Jul 18;5(7):e2222721. doi: 10.1001/jamanetworkopen.2022.22721 (PMC9295000; doi:10.1001/jamanetworkopen.2022.22721)
Supplement: Supplement. — eFigure 1. Liver-Related Toxic Effects in Placebo-Controlled Trials and Trials With Active Comparator eFigure 2. Serious Adverse Events in Placebo-Controlled Trials and in Trials With Active Comparator eFigure 3. Grade 3 or Higher Adverse Events in Placebo-Controlled Trials and Trials With Active Comparator eFigure 4. Risk of Bias 2 Assessment eTable. MINORS Assessment eFigure 5. GRADE of Evidence Assessment [file jamanetwopen-e2222721-s001.pdf]

Supplemental Online Content

Griffiths CD, Zhang B, Tywonek K, Meyers BM, Serrano PE. Toxicity profiles of systemic therapies for advanced hepatocellular carcinoma: a systematic review and meta-analysis. *JAMA Netw Open.* 2022;5(7):e2222721. doi:10.1001/jamanetworkopen.2022.22721

eFigure 1. Liver-Related Toxic Effects in Placebo-Controlled Trials and Trials With Active Comparator

eFigure 2. Serious Adverse Events in Placebo-Controlled Trials and in Trials With Active Comparator

eFigure 3. Grade 3 or Higher Adverse Events in Placebo-Controlled Trials and Trials With Active Comparator

eFigure 4. Risk of Bias 2 Assessment

eTable. MINORS Assessment

eFigure 5. GRADE of Evidence Assessment

This supplemental material has been provided by the authors to give readers additional information about their work.

© 2022 Griffiths CD et al. *JAMA Network Open.*

eFigure 1. Liver-Related Toxic Effects in Placebo-Controlled Trials and Trials With Active Comparator

A. Placebo-Controlled Trials

Study or Subgroup	Experimental Events	Experimental Total	Control Events	Control Total	Weight	Risk Ratio M-H, Random, 95% CI
1.1.1 TKI Therapy vs. Placebo						
Abou-Alfa et al., #11 2018	94	467	25	237	12.3%	1.91 [1.26, 2.88]
Bruix et al., #10 2017	59	374	27	193	12.1%	1.13 [0.74, 1.72]
Kang et al., #51 2015	94	133	40	68	16.1%	1.20 [0.96, 1.51]
Kudo et al., #53 2011	20	229	5	227	4.8%	3.97 [1.51, 10.38]
Llovet et al., #54 2013	42	263	16	132	9.9%	1.32 [0.77, 2.25]
Llovet et al., #8 2008	1	299	0	303	0.6%	3.04 [0.12, 74.33]
Rimassa et al., #56 2018	19	225	11	114	7.3%	0.88 [0.43, 1.78]
Santoro et al., #57 2013	8	71	1	36	1.3%	4.06 [0.53, 31.19]
Zhu et al., #58 2014	50	362	12	184	8.8%	2.12 [1.16, 3.88]
Zhu et al., #59 2015 (I)	56	277	66	276	14.3%	0.85 [0.62, 1.16]
Zhu et al., #61 2019	4	197	0	95	0.7%	4.36 [0.24, 80.23]
Subtotal (95% CI)		2897		1865	88.2%	1.38 [1.06, 1.79]
Total events	447		203			
Heterogeneity: Tau² = 0.08; Chi² = 22.77, df = 10 (P = 0.01); I² = 56%						
Test for overall effect: Z = 2.42 (P = 0.02)						
1.1.2 PD-1/PD-L1 Therapy vs. Placebo						
Finn et al., #14 2020 (I)	79	278	21	135	11.8%	1.83 [1.18, 2.82]
Subtotal (95% CI)		278		135	11.8%	1.83 [1.18, 2.82]
Total events	79		21			
Heterogeneity: Not applicable						
Test for overall effect: Z = 2.71 (P = 0.007)						
Total (95% CI)		3175		2000	100.0%	1.43 [1.12, 1.82]
Total events	526		224			
Heterogeneity: Tau² = 0.08; Chi² = 25.58, df = 11 (P = 0.007); I² = 57%						
Test for overall effect: Z = 2.84 (P = 0.005)						
Test for subgroup differences: Chi² = 1.17, df = 1 (P = 0.28), I² = 14.5%						

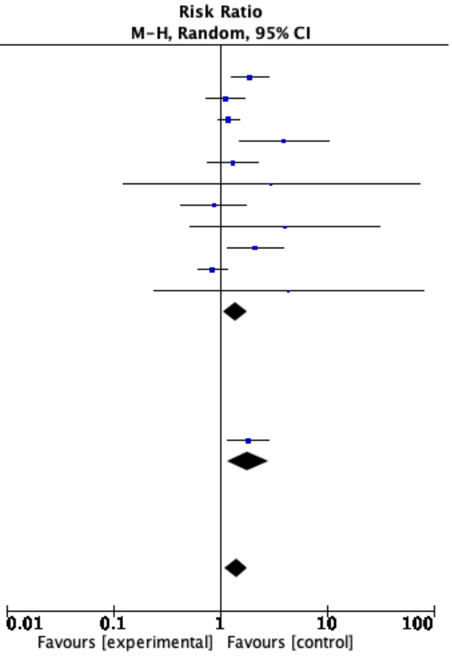

B. Trials With Active Comparator

Study or Subgroup	Experimental Events	Experimental Total	Control Events	Control Total	Weight	Risk Ratio M-H, Random, 95% CI
4.1.1 TKI vs. Sorafenib						
Cainap et al. 2015	180	514	174	521	32.4%	1.05 [0.89, 1.24]
Cheng et al. 2013	77	526	65	542	10.9%	1.22 [0.90, 1.66]
Cheng et al. 2016	42	79	33	83	9.2%	1.34 [0.96, 1.87]
Johnson et al. 2013	34	577	29	578	4.6%	1.17 [0.73, 1.90]
Koeberle et al. 2016	7	59	12	46	1.5%	0.45 [0.19, 1.06]
Kudo et al. 2018	55	476	61	475	8.9%	0.90 [0.64, 1.27]
Palmer et al. 2018	17	62	4	31	1.1%	2.13 [0.78, 5.78]
Zhu et al. 2015 (2)	74	362	79	358	12.9%	0.93 [0.70, 1.23]
Subtotal (95% CI)		2655		2634	81.5%	1.06 [0.92, 1.24]
Total events	486		457			
Heterogeneity: Tau² = 0.01; Chi² = 10.28, df = 7 (P = 0.17); I² = 32%						
Test for overall effect: Z = 0.81 (P = 0.42)						
4.1.2 PD-1/L1+TKI vs. Other Active Agent						
Lee et al. 2019	4	60	3	58	0.5%	1.29 [0.30, 5.51]
Subtotal (95% CI)		60		58	0.5%	1.29 [0.30, 5.51]
Total events	4		3			
Heterogeneity: Not applicable						
Test for overall effect: Z = 0.34 (P = 0.73)						
4.1.4 PD-1+TKI vs. Sorafenib						
Cheng et al. 2015 (2) (I)	22	53	22	55	5.1%	1.04 [0.66, 1.64]
Cheng et al. 2015 (2) (II)	28	54	22	55	6.2%	1.30 [0.86, 1.96]
Finn et al. 2020 (IMBRAVE150)	60	336	30	165	6.7%	0.98 [0.66, 1.46]
Subtotal (95% CI)		443		275	18.0%	1.10 [0.86, 1.40]
Total events	110		74			
Heterogeneity: Tau² = 0.00; Chi² = 1.00, df = 2 (P = 0.61); I² = 0%						
Test for overall effect: Z = 0.75 (P = 0.45)						
Total (95% CI)		3158		2967	100.0%	1.07 [0.96, 1.19]
Total events	600		534			
Heterogeneity: Tau² = 0.00; Chi² = 11.41, df = 11 (P = 0.41); I² = 4%						
Test for overall effect: Z = 1.25 (P = 0.21)						
Test for subgroup differences: Chi² = 0.11, df = 2 (P = 0.95), I² = 0%						

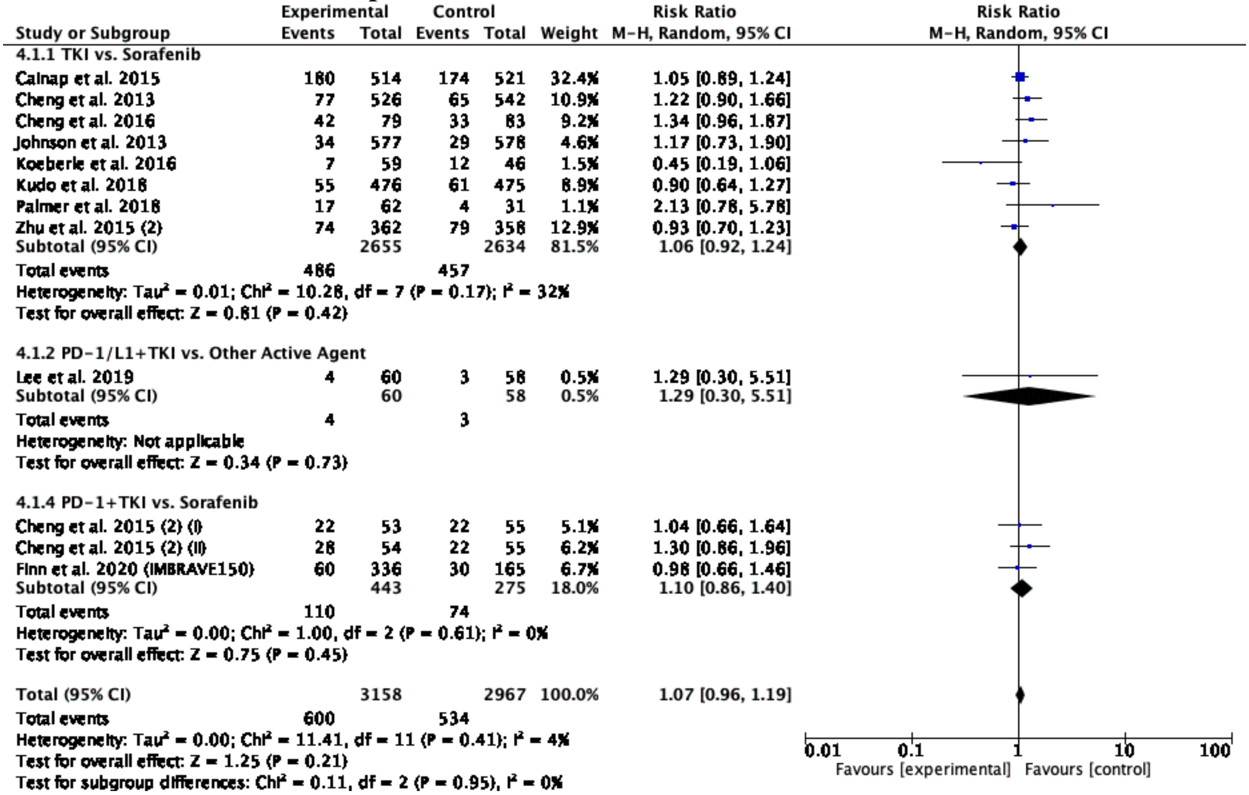

Lee et al., 2019 compares atezolizumab + bevacizumab to atezolizumab. The remaining comparators are Sorafenib.

© 2022 Griffiths CD et al. *JAMA Network Open.*

eFigure 2. Serious Adverse Events in Placebo Controlled Trials and in Trials With Active Comparator

A. Placebo-Controlled Trials

Study or Subgroup	Treatment Events	Treatment Total	Control Events	Control Total	Weight	Risk Ratio M-H, Random, 95% CI
**2.1.1 TKI vs Placebo**						
Bruix et al. 2017	166	374	90	193	11.7%	0.95 [0.79, 1.15]
Cheng et al. 2009	71	149	34	75	8.8%	1.05 [0.78, 1.42]
Kang et al. 2015	63	134	17	68	5.8%	1.88 [1.20, 2.95]
Kudo et al. 2011	42	229	25	227	5.7%	1.67 [1.05, 2.64]
Llovet et al. 2013	165	261	75	131	12.0%	1.10 [0.93, 1.31]
Ramissa et al. 2018	103	225	51	114	10.1%	1.02 [0.80, 1.31]
Santoro et al. 2013 ()	24	69	14	36	4.8%	0.89 [0.53, 1.51]
Zhu et al. 2014	173	362	65	184	10.7%	1.35 [1.08, 1.69]
Zhu et al. 2015 (1)	100	277	79	276	10.2%	1.26 [0.99, 1.61]
Zhu et al. 2015 (2)	210	362	196	538	12.9%	1.59 [1.38, 1.84]
Zhu et al. 2019	68	197	28	95	7.3%	1.17 [0.81, 1.69]
Subtotal (95% CI)		2639		1937	100.0%	1.22 [1.06, 1.40]
Total events	1185		674			
Heterogeneity: $Tau^2 = 0.03$; $Chi^2 = 31.82$, df = 10 (P = 0.0004); $I^2 = 69\%$						
Test for overall effect: Z = 2.83 (P = 0.005)						
Total (95% CI)		2639		1937	100.0%	1.22 [1.06, 1.40]
Total events	1185		674			
Heterogeneity: $Tau^2 = 0.03$; $Chi^2 = 31.82$, df = 10 (P = 0.0004); $I^2 = 69\%$						
Test for overall effect: Z = 2.83 (P = 0.005)						
Test for subgroup differences: Not applicable						

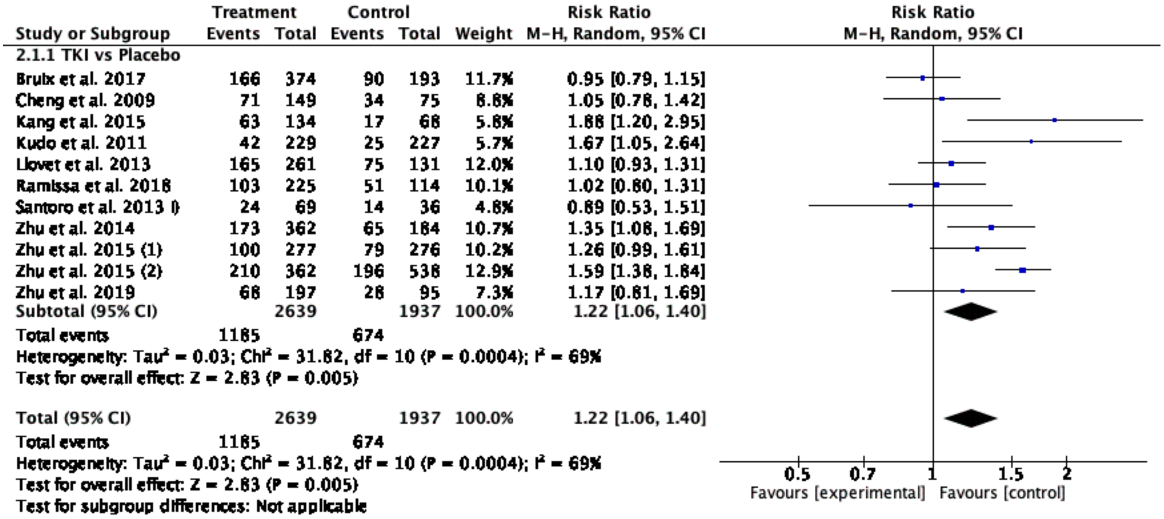

B. Trials With Active Comparator

Study or Subgroup	Experimental Events	Experimental Total	Control Events	Control Total	Weight	Risk Ratio M-H, Random, 95% CI
**4.2.1 TKI vs Sorafenib**						
Cainap et al. 2015	267	510	200	519	17.6%	1.36 [1.19, 1.56]
Cheng et al. 2013	0	526	0	542		Not estimable
Cheng et al. 2015 (1)	29	63	18	32	5.1%	0.82 [0.55, 1.23]
Cheng et al. 2016	40	71	34	83	7.1%	1.38 [0.99, 1.91]
Johnson et al. 2013	310	575	276	575	19.4%	1.12 [1.00, 1.26]
Kudo et al. 2018	205	476	144	475	14.9%	1.42 [1.20, 1.69]
Zhu et al. 2015 (2)	0	0	0	0		Not estimable
Subtotal (95% CI)		2221		2226	64.1%	1.24 [1.07, 1.44]
Total events	851		672			
Heterogeneity: $Tau^2 = 0.02$; $Chi^2 = 11.55$, df = 4 (P = 0.02); $I^2 = 65\%$						
Test for overall effect: Z = 2.93 (P = 0.003)						
**4.2.2 ICI vs Sorafenib**						
Finn et al. 2020 (IMBRAVE150)	125	329	48	156	9.1%	1.23 [0.94, 1.62]
Yau et al. 2021	43	371	39	372	5.1%	1.11 [0.73, 1.66]
Subtotal (95% CI)		700		528	14.2%	1.19 [0.95, 1.50]
Total events	168		87			
Heterogeneity: $Tau^2 = 0.00$; $Chi^2 = 0.20$, df = 1 (P = 0.66); $I^2 = 0\%$						
Test for overall effect: Z = 1.53 (P = 0.13)						
**4.2.3 TKI + Sorafenib vs Sorafenib**						
Koeberle et al. 2016	28	60	14	46	3.5%	1.53 [0.92, 2.56]
Zhu et al. 2015 (2)	210	362	195	358	18.2%	1.07 [0.94, 1.21]
Subtotal (95% CI)		422		404	21.6%	1.17 [0.85, 1.61]
Total events	238		209			
Heterogeneity: $Tau^2 = 0.03$; $Chi^2 = 1.85$, df = 1 (P = 0.17); $I^2 = 46\%$						
Test for overall effect: Z = 0.98 (P = 0.33)						
Total (95% CI)		3343		3158	100.0%	1.21 [1.09, 1.34]
Total events	1257		968			
Heterogeneity: $Tau^2 = 0.01$; $Chi^2 = 16.82$, df = 8 (P = 0.03); $I^2 = 52\%$						
Test for overall effect: Z = 3.64 (P = 0.0003)						
Test for subgroup differences: $Chi^2 = 0.16$, df = 2 (P = 0.92), $I^2 = 0\%$						

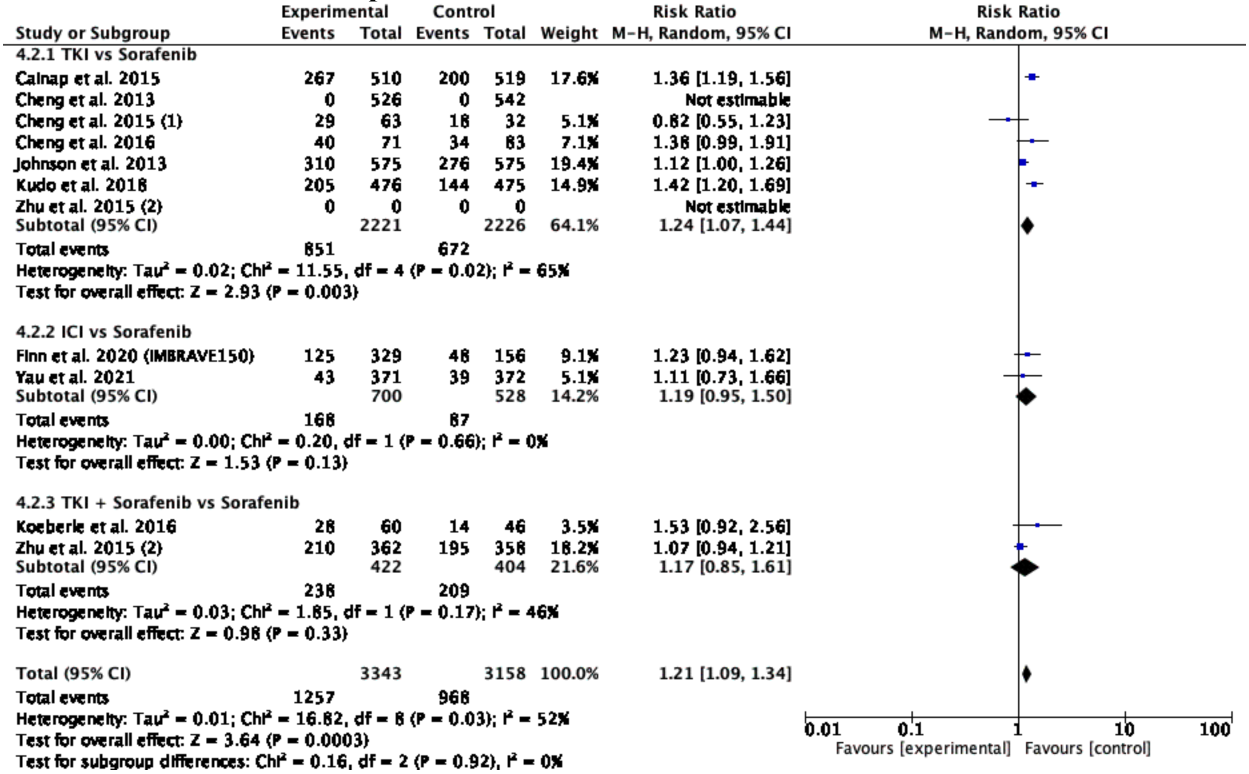

© 2022 Griffiths CD et al. *JAMA Network Open.*

eFigure 3. Grade 3 or Higher Adverse Events in Placebo-Controlled Trials and Trials With Active Comparator

A, Placebo-Controlled Trials

Study or Subgroup	Experimental Events	Experimental Total	Control Events	Control Total	Weight	Risk Ratio M-H, Random, 95% CI
2.2.1 TKI vs Placebo						
Abou-Alfa et al. 2018	320	470	86	237	18.3%	1.88 [1.57, 2.25]
Llovet et al. 2008	45	297	32	302	12.2%	1.43 [0.94, 2.18]
Llovet et al. 2013	178	261	32	131	15.0%	2.79 [2.04, 3.82]
Ramissa et al. 2018	155	225	63	114	18.1%	1.25 [1.03, 1.50]
Zhu et al. 2014	257	362	96	184	18.9%	1.36 [1.17, 1.59]
Subtotal (95% CI)		1615		968	82.5%	1.65 [1.27, 2.14]
Total events	955		309			
Heterogeneity: $Tau^2 = 0.07$; $Chi^2 = 27.74$, $df = 4$ ($P < 0.0001$); $I^2 = 86\%$						
Test for overall effect: $Z = 3.75$ ($P = 0.0002$)						
2.2.2 PD-1 vs Placebo						
Finn et al. 2020 (KEYNOTE240)	147	278	62	135	17.5%	1.15 [0.93, 1.43]
Subtotal (95% CI)		278		135	17.5%	1.15 [0.93, 1.43]
Total events	147		62			
Heterogeneity: Not applicable						
Test for overall effect: $Z = 1.29$ ($P = 0.20$)						
Total (95% CI)		1893		1103	100.0%	1.55 [1.23, 1.95]
Total events	1102		371			
Heterogeneity: $Tau^2 = 0.07$; $Chi^2 = 33.78$, $df = 5$ ($P < 0.00001$); $I^2 = 85\%$						
Test for overall effect: $Z = 3.68$ ($P = 0.0002$)						
Test for subgroup differences: $Chi^2 = 4.31$, $df = 1$ ($P = 0.04$), $I^2 = 76.8\%$						

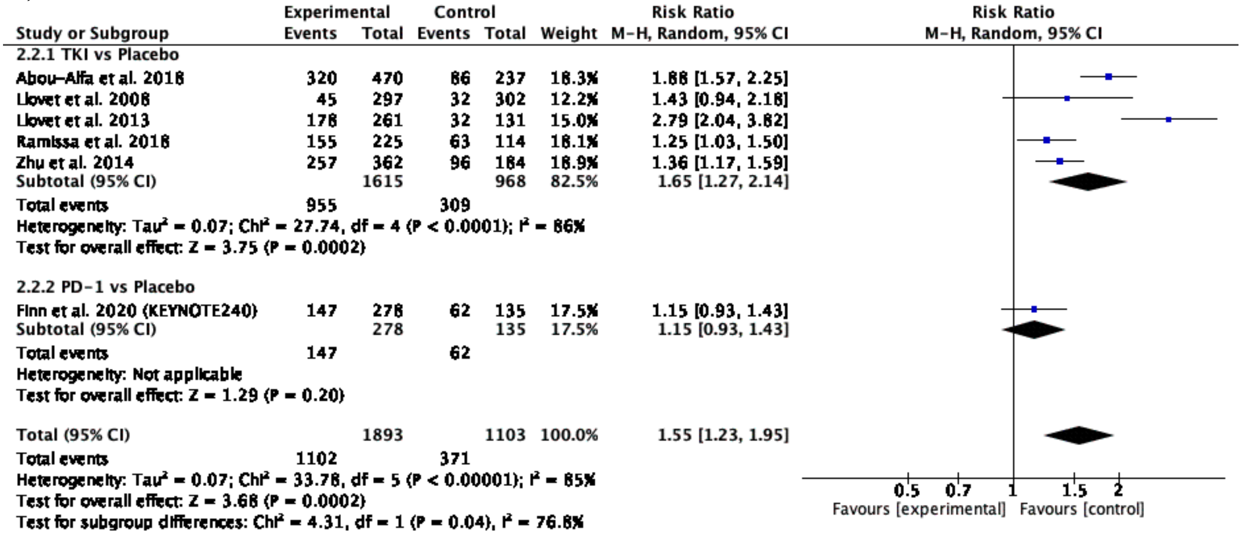

B. Trials With Active Comparator

Study or Subgroup	Experimental Events	Experimental Total	Control Events	Control Total	Weight	Risk Ratio M-H, Random, 95% CI
4.3.1 TKI vs Sorafenib						
Cainap et al. 2015	435	510	289	512	14.2%	1.51 [1.39, 1.64]
Cheng et al. 2013	432	526	402	542	14.6%	1.11 [1.04, 1.18]
Cheng et al. 2015 (1)	35	63	27	34	8.7%	0.70 [0.53, 0.93]
Kudo et al. 2018	357	476	316	475	14.2%	1.13 [1.04, 1.22]
Palmer et al. 2018	42	62	28	31	10.7%	0.75 [0.61, 0.92]
Zhu et al. 2015 (2)	315	362	258	355	14.4%	1.20 [1.11, 1.29]
Subtotal (95% CI)		1999		1949	76.8%	1.07 [0.92, 1.24]
Total events	1616		1320			
Heterogeneity: $Tau^2 = 0.03$; $Chi^2 = 70.97$, $df = 5$ ($P < 0.00001$); $I^2 = 93\%$						
Test for overall effect: $Z = 0.89$ ($P = 0.38$)						
4.3.2 ICI vs Sorafenib						
Finn et al. 2020 (IMBRAVE150)	201	329	95	156	12.4%	1.00 [0.86, 1.17]
Subtotal (95% CI)		329		156	12.4%	1.00 [0.86, 1.17]
Total events	201		95			
Heterogeneity: Not applicable						
Test for overall effect: $Z = 0.04$ ($P = 0.97$)						
4.3.3 TKI + Sorafenib vs Sorafenib						
Koeberle et al. 2016	53	60	33	46	10.9%	1.23 [1.00, 1.51]
Subtotal (95% CI)		60		46	10.9%	1.23 [1.00, 1.51]
Total events	53		33			
Heterogeneity: Not applicable						
Test for overall effect: $Z = 2.01$ ($P = 0.04$)						
Total (95% CI)		2388		2151	100.0%	1.08 [0.95, 1.23]
Total events	1870		1448			
Heterogeneity: $Tau^2 = 0.03$; $Chi^2 = 74.74$, $df = 7$ ($P < 0.00001$); $I^2 = 91\%$						
Test for overall effect: $Z = 1.21$ ($P = 0.23$)						
Test for subgroup differences: $Chi^2 = 2.50$, $df = 2$ ($P = 0.29$), $I^2 = 20.1\%$						

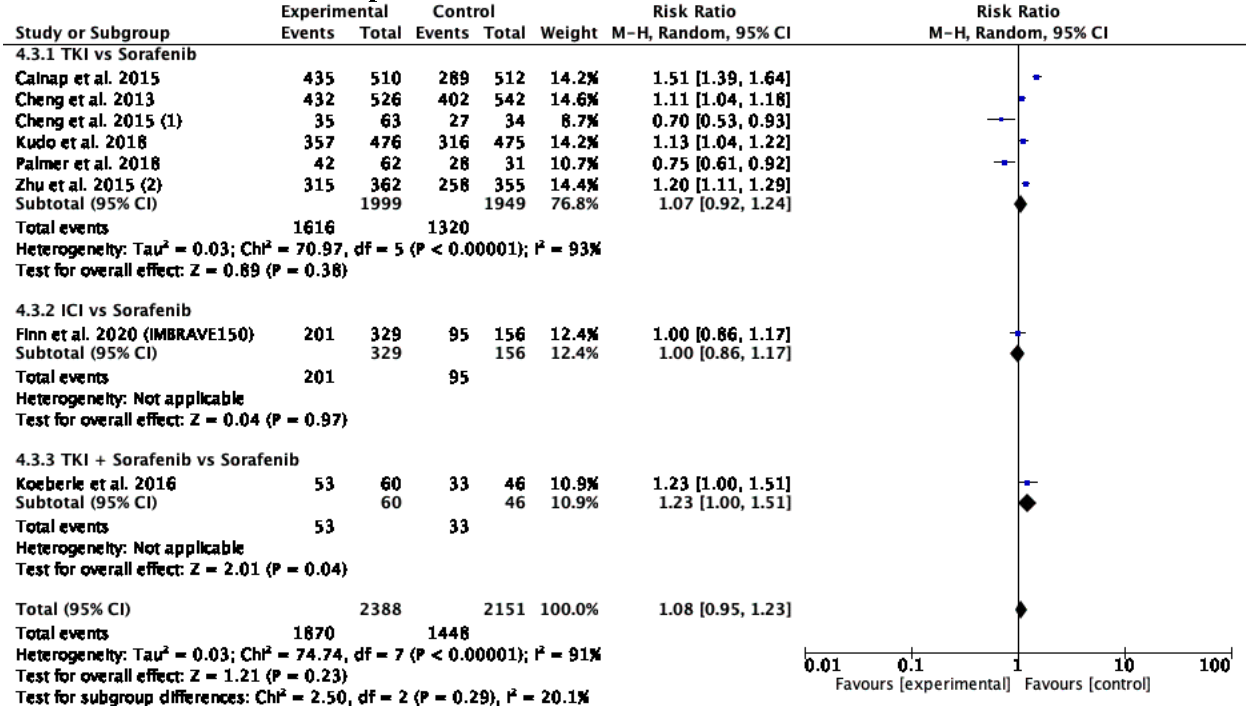

© 2022 Griffiths CD et al. *JAMA Network Open.*

eFigure 4. Risk of Bias 2 Assessment

Per-protocol	Unique ID	Study ID	Experimental	Comparator	Outcome	Weight	D1	D2	D3	D4	D5	Overall
	C1	AbouAlfa	Cabozantinib	Placebo	Adverse Events	NA	+	+	+	+	+	+
	C2	Brouix	Regorafenib	Placebo	Adverse Events	1	+	+	+	+	+	+
	C3	Cainap	Linifanib	Sorafenib	Adverse Events	1	+	+	+	!	+	!
	C4	Cheng2009	Sorafenib	Placebo	Adverse Events	1	+	+	+	+	+	+
	C5	Cheng2015	Sunitinib	Sorafenib	Adverse Events	1	+	+	+	!	+	!
	C6	Cheng2015 1	Nintedanib	Sorafenib	Adverse Events	1	+	+	+	!	+	!
	C7	Cheng2015 2	Tigatuzumab	Sorafenib	Adverse Events	1	+	+	+	!	+	!
	C8	Cheng 2016	Dovitinib	Sorafenib	Adverse Events	1	+	+	+	!	+	+
	C9	Finn Key	Pembrolizumab	Placebo	Adverse Events	1	+	+	+	+	+	+
	C10	Finn Imbrave	Atezolizumab/Bevacizumab	Sorafenib	Adverse Events	1	+	+	+	+	+	+
	C11	Johnso2013	Brivanib	Sorafenib	Adverse Events	1	+	+	+	+	+	+
	C12	Kang2015	Axitinib	Placebo	Adverse Events	1	+	+	+	+	+	+
	C13	Kaseb2020	Nivolumab	Nivolumab+Ipilimumab	Adverse Events	1	+	+	+	!	+	!
	C14	Kelley2020	Durvulamab + Tremulimuma	Monotherapy	Adverse Events	1	+	+	+	!	+	!
	C15	Koeberle 2016	Sorafenib/Everolimus	Sorafenib	Adverse Events	1	+	+	+	!	+	!
	C16	Kudo2011	Sorafenib	Placebo	Adverse Events	1	+	+	+	+	+	+
	C17	Kudo2018	Lenvatinib	Sorafenib	Adverse Events	1	+	+	+	!	+	!
	C18	Llovet2008	Sorafenib	Placebo	Adverse Events	1	+	+	+	+	+	+
	C19	Llovet2013	Brivanib	Placebo	Adverse Events	1	+	+	+	+	+	+
	C20	Lee2019	Atezolizumab/Bevacizumab	Atezolizumab	Adverse Events	1	+	+	+	!	+	!
	C21	Palmer2018	Nintedanib	Sorafenib	Adverse Events	1	+	+	+	!	+	!
	C22	Rimassa2018	Tivantinib	Placebo	Adverse Events	1	+	+	+	+	+	+
	C23	Santoro2013	Tivantinib	Placebo	Adverse Events	1	+	+	+	+	+	+
	C24	Yau2020	Nivo/Ipi	Placebo	Adverse Events	1	+	+	+	!	+	!
	C25	Zhu2014	Everolimus	Placebo	Adverse Events	1	+	+	+	+	+	+
	C26	Zhu2015 1	Ramucirumab	Placebo	Adverse Events	1	+	+	+	+	+	+
	C27	Zhu2015 2	Sorafenib/Erlotinib	Sorafenib/Placebo	Adverse Events	1	+	+	+	+	+	+
	C28	Zhu2019	Ramucirumab	Placebo	Adverse Events	1	+	+	+	+	+	+

- + Low risk
- ! Some concerns
- - High risk

- D1 Randomisation process
- D2 Deviations from the intended interventions
- D3 Missing outcome data
- D4 Measurement of the outcome
- D5 Selection of the reported result

© 2022 Griffiths CD et al. *JAMA Network Open.*

eTable. MINORS Assessment

	Marron et al., 2022	Finn et al., 2020 (iii)
**A clearly stated aim**	2	2
**Inclusion of consecutive patients**	0	2
**Prospective collection of data**	2	2
**Appropriate endpoints**	2	2
**Unbiased assessment of endpoints**	2	2
**Appropriate follow up**	2	2
**Loss to follow up <5%**	2	2
**Prospective calculation of sample size**	2	2
**Total**	14/16	16/16

© 2022 Griffiths CD et al. *JAMA Network Open.*

eFigure 5. GRADE of Evidence Assessment

[illegible]

Certainty assessment							№ of patients		Effect		Certainty	Importance
**№ of studies**	**Study design**	**Risk of bias**	**Inconsistency**	**Indirectness**	**Imprecision**	**Other considerations**	**TKIs**	**ICIs**	**Relative (95% CI)**	**Absolute (95% CI)**		
**IV Liver**												
23	randomised trials	not serious	not serious	serious[a]	not serious	none	1688/8040 (21.0%)	301/1075 (28.0%)	**Proportion 0.23** (0.18 to 0.28)	**-- per 1,000** (from -- to --)	⊕⊕⊕◯ Moderate	
**IV serious AEs**												
23	randomised trials	not serious	not serious	serious	not serious	none	2998/6517 (46.0%)	304/1266 (24.0%)	**Proportion 0.41** (0.34 to 0.48)	**-- per 1,000** (from -- to --)	⊕⊕⊕◯ Moderate	
**IV grade 3<**												
20	randomised trials	not serious	not serious	serious[a]	not serious	none	3908/5665 (69.0%)	555/1587 (35.0%)	**Proportion 0.56** (0.46 to 0.67)	**-- per 1,000** (from -- to --)	⊕⊕⊕◯ Moderate	

CI: confidence interval

Explanations

a. Proportions largely compared between different studies

.

© 2022 Griffiths CD et al. *JAMA Network Open.*
